# Origin and Consequences of the Relationship between Protein Mean and Variance

**DOI:** 10.1371/journal.pone.0102202

**Published:** 2014-07-25

**Authors:** Francesco Luigi Massimo Vallania, Marc Sherman, Zane Goodwin, Ilaria Mogno, Barak Alon Cohen, Robi David Mitra

**Affiliations:** 1 Center for Genome Sciences and Systems Biology, Department of Genetics, Washington University School of Medicine, St. Louis, Missouri, United States of America; 2 Program in Computational and Systems Biology, Washington University School of Medicine, St. Louis, Missouri, United States of America; 3 Program in Computational and Molecular Biophysics, Washington University School of Medicine, St. Louis, Missouri, United States of America; Spanish National Research Council (CSIC), Spain

## Abstract

Cell-to-cell variance in protein levels (noise) is a ubiquitous phenomenon that can increase fitness by generating phenotypic differences within clonal populations of cells. An important challenge is to identify the specific molecular events that control noise. This task is complicated by the strong dependence of a protein's cell-to-cell variance on its mean expression level through a power-law like relationship (σ^2^∝μ^1.69^). Here, we dissect the nature of this relationship using a stochastic model parameterized with experimentally measured values. This framework naturally recapitulates the power-law like relationship (σ^2^∝μ^1.6^) and accurately predicts protein variance across the yeast proteome (r^2^ = 0.935). Using this model we identified two distinct mechanisms by which protein variance can be increased. Variables that affect promoter activation, such as nucleosome positioning, increase protein variance by changing the exponent of the power-law relationship. In contrast, variables that affect processes downstream of promoter activation, such as mRNA and protein synthesis, increase protein variance in a mean-dependent manner following the power-law. We verified our findings experimentally using an inducible gene expression system in yeast. We conclude that the power-law-like relationship between noise and protein mean is due to the kinetics of promoter activation. Our results provide a framework for understanding how molecular processes shape stochastic variation across the genome.

## Introduction

Stochastic fluctuations in the biochemical processes that underlie gene expression produce cell-to-cell variation in protein levels, or “noise” [1]–[3]. Noise performs several biological functions. In unicellular organisms, noise improves fitness by generating phenotypic differences within clonal populations of cells, thus enabling a rapid response to fluctuating environments [4]–[6]. In multi-cellular organisms, noise plays a role in development, allowing identical progenitor cells to acquire distinct fates [7]–[9]. Because of its functional importance, a fundamental goal is to identify and dissect the molecular mechanisms that generate and control noise.

Single-cell studies have connected pathway-specific (extrinsic) and gene-specific (intrinsic) factors to changes in protein variance [2], [10], [11]. These factors include the rate of transcript elongation [12], the presence of a TATA-box [2], [4], [13], [14], nucleosome positioning at the promoter sequence [2], [15]–[18], fluctuating mRNA levels [19], translation rate [18], [20], [21], pathway-dependent fluctuations [11], [19], and asymmetric partitioning at cell division [22]. However, it is unclear whether any of these processes evolved specifically to produce high levels of protein variance, or whether the observed variance is only a consequence of selective pressure on protein mean levels. This issue is complicated by the strong dependence of cell-to-cell protein variance on mean protein levels [11], [19], [20]. Several studies have revealed that a protein's cell-to-cell variance is linearly related to its mean expression level when plotted on a log-log scale, suggesting this relationship can be approximated by a power-law (σ^2^∝µ^j^) [19], [23], [24]. This relationship is of paramount importance for investigations into the evolutionary origins and consequences of noise, because it allows to correctly normalize protein variances to identify proteins that display unexpectedly high single-cell variance given their mean levels. Although this relationship has been noted previously, two important questions have not yet been resolved. First, how does the process of gene expression specify this power-law relationship and consequently protein variance? Secondly, which molecular processes regulating gene expression have evolved to produce substantially higher protein variance than would be expected given the observed relationship?

To answer these questions, we analyzed a dataset of protein variance using a stochastic model of gene expression parameterized with experimentally measured kinetic rates. This model recapitulated the relationship (σ^2^∝μ^1.6^) between mean and variance and accurately predicted protein variance on a proteome-wide scale (r^2^ = 0.935). We find that this result is achieved under a general regime of promoter kinetics across the yeast genome characterized by slow promoter activation followed by rapid inactivation, resulting in mRNA production that is nearly a Poisson process (σ^2^∝μ^1.1^). However, the small non-linearity between RNA mean and variance is amplified during protein production, reproducing the observed power law. By further analyzing this model, we found that the kinetics of promoter activation dictate the exponent of the power-law. This finding allowed us to identify two distinct classes of processes that influence noise. Variables that influence processes downstream of promoter activation, such as the synthesis and degradation of mRNA and protein, increase variance by increasing mean levels, which then causes an concomitant change in protein variance in accordance with the power law. In contrast, variables that reduce the rate of promoter activation, such as promoter-positioned nucleosomes, increase variance by increasing the exponent of the power-law-like relationship linking protein mean and variance. Only the latter class of mechanisms generate protein variances that are significantly higher than expected from protein mean levels.

In support of these conclusions, we performed experiments demonstrating that changing the rate of promoter activation, but not the rate of protein translation, modulates the exponent of the power-law and consequently the scaling between variance and mean. By providing a mechanistic interpretation of the power-law-like relationship, our work provides the framework to achieve a better understanding of the molecular processes that lead to cell-to-cell variation in gene expression.

## Results

### Protein mean and variance are connected by a power-law-like relationship

**Figure 1:**
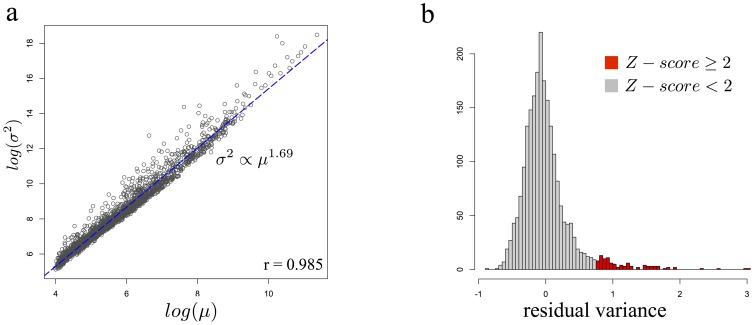
Relationship between mean and variance in protein expression. a) Protein mean and variance values in *S. cerevisiae* plotted against each other in log-scale in arbitrary fluorescence, with corresponding Pearson's correlation coefficient. b) Distribution of residual variance values across the *S. cerevisiae* dataset. Red bars indicate residual variance value with Z-scores over 2 standard deviations from the mean.

To characterize the relationship between mean protein levels and cell-to-cell protein variance across the yeast genome, we analyzed a published dataset consisting of ∼2200 *S. cerevisiae* GFP fusion strains for which protein levels had been measured at a single-cell resolution by flow-cytometry [13]. This dataset serves as a starting point to examine global trends between protein mean and variance as it represents an unbiased sampling of the yeast proteome. First, we performed a log-log regression analysis of cell-to-cell protein variance as a function of the mean protein levels and observed a power-law-like relationship with an exponent of 1.69 (Figure 1a), in agreement with previous findings [23]. Ninety-seven percent of protein variance across the proteome can be explained solely by mean levels through this relationship, indicating that highly expressed genes naturally exhibit high cell-to-cell variation whereas genes expressed at low levels are more uniformly expressed across different cells. Although the residual fraction of protein variance not explained by the power-law accounts for only 3% of the total variation, we found that, for certain genes, it increased protein variance up to 20-fold higher than expected (Figure 1b). In contrast, very few genes displayed smaller protein variances than expected given mean levels, as we observed, at most, a 2-fold reduction (see Figure 1b). Taken together, these results indicate that for most genes, protein variance is largely explained by the protein mean through a power-law-like relationship, except for a few notable cases in which protein variances are increased substantially beyond their expected values.

### A stochastic model of gene expression recapitulates the power-law-like relationship between protein mean and variance

We next sought to understand the molecular origin of the relationship between protein mean and variance. One hypothesis is that this relationship originates purely as a consequence of stochasticity in the steps underlying gene expression [19]. Alternatively, this relationship could result from mechanisms that are independent of expression, such as asymmetric partitioning of protein and RNA molecules at cell division [22] or pathway-dependent fluctuations in trans-acting factors [11].

**Figure 2:**
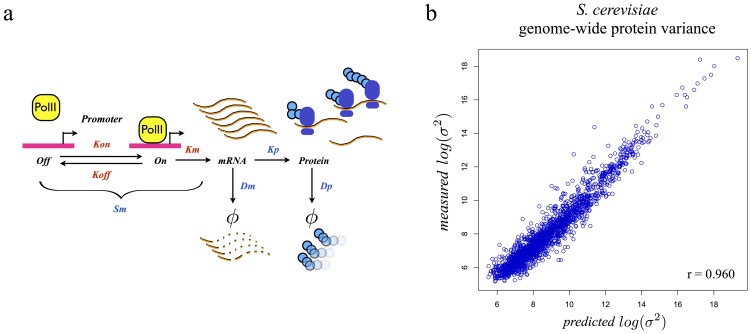
Stochastic model of gene expression: a) Schematic representation of the model. Each step transition is determined by a rate constant. Promoter activation and inactivation occur at *Kon* and *Koff* rates respectively. When active, a promoter is transcribed at *Km* rate into an mRNA molecule. The mRNA molecule can then be either degraded at *Dm* rate or translated at *Kp* rate into a protein. The protein molecule can then be degraded at rate *Dp. Kon, Koff*, and *Km* determine the synthesis rate of mRNA, or *Sm*. Blue indicates that the parameter has been empirically measured or calculated across the dataset, red indicates that the parameter has been simplified or fit across the dataset b) Model performance in predicting protein variance in *S. cerevisiae*. Each point represents a single GFP fusion strain. Data is displayed in log-scale (linear scale r = 0.836).

To distinguish between these two hypotheses, we tested whether a stochastic model based only on the processes involved in gene expression could recapitulate the observed power-law relationship. We applied a model [25] that describes cell-to-cell protein variance at steady-state as a function of kinetic parameters for promoter activation/inactivation events and mRNA and protein production/degradation (Figure 2a, Figure S1). For most parameter values, we used empirical measurements (see Supporting Information S1, section 1.2). This was not possible, however, for the rates of promoter activation and inactivation, which have only been measured in a few genes [26]. Since no high-throughput methods exist for measuring rates of promoter activation and inactivation, we assumed that the promoter kinetics would be similar across the genome and fit their values from the data (Supporting Information S1, section 1.3). The model converged to a regime in which promoter activation is an infrequent event that is quickly followed by promoter inactivation, a result supported by published experimental data [23] (Supporting Information S1, section 1.4, and Figure S1). We obtained a rate of promoter activation (*Kon*) of 0.59 min^−1^, a value that agrees with empirically measured activation rate for the *GLT1* gene in yeast (1.3±0.72 min^−1^) [26]. Using this value for *Kon*, the model naturally generates a power-law-like relationship between mean and variance that is similar to the one observed empirically (modeled relationship: σ^2^∝μ^1.60^, observed relationship: σ^2^∝μ^1.69^). Furthermore, our framework correctly predicts protein variance across the genome (log space r = 0.962, p<2.2*10^−16^; linear space r = 0.839, p<2.2*10^−16^, Figure 2b). We tested for over-fitting by performing 2-fold cross-validation 100 times and again found good agreement (r = 0.957±0.018, p<2.2*10^−16^). Taken together, these results support the validity of our model and suggest that the power-law relationship between protein mean and variance depends solely on the kinetics of the processes that underlie gene expression.

### The power-law-like relationship between protein variance and mean depends on promoter kinetics

**Figure 3:**
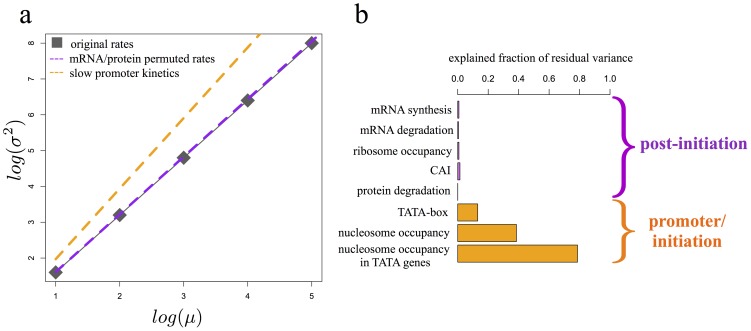
Promoter kinetics but not mRNA and protein synthesis and degradation rates modulate the relationship between mean and variance. a) Predicted relationship between mean and variance using original model with original parameter set (grey squares), original model with permuted sets of kinetic rates for mRNA/protein synthesis and degradation (purple), and slow promoter kinetics model with original parameter set (orange). b) Fraction of residual variance explained (r^2^) by sources of noise operating at the promoter/initiation level (orange) or at a post-initiation level (purple).

We next sought to determine which of the processes involved in gene expression determine the exponent of the power-law-like relationship. Using our biophysical model, we randomly sampled transcription and translation rates, as well as degradation rates of mRNA and protein, while maintaining the same promoter activation regime we identified above (*Kon* = 0.59 min^−1^<< *Koff*). Virtually all permutations resulted in a power-law-like relationship between mean and variance that was nearly identical to the one observed experimentally (exponent  = 1.612±5.9*10^−3^, 1000 permutations, Figure 3a). This result indicates that, when *Kon* << *Koff*, the exact form of the power-law-like relationship between mean and variance is independent of the rates of transcription, translation, and protein and mRNA degradation.

In contrast, we found that the exponent of the power law was strongly dependent on promoter kinetics. Using the same modeling framework, we changed the parameters governing promoter transitions to enforce a slow kinetics regime (*Kon* and *Koff* << *Km,Dm,Kp,Dp*). We found that protein mean and variance followed a quadratic relationship (exponent  = 1.97, Figure 3a), which differs substantially from our previous results and the observed power-law. Taken together these results suggest that the power-law relationship between protein mean and cell-to-cell variance is dictated by the kinetics of promoter activation, and is largely insensitive to downstream steps.

### The relationship between protein mean and variance identifies different sources of variance

A strong prediction of our model is that perturbations that affect processes downstream of promoter activation should increase noise only through changes in mean protein level, which will then increase protein variance following the power law. In contrast, perturbations which affect the kinetics of promoter activation should increase protein variance by modulating the relationship between protein mean and variance. As a result, this class of perturbations are expected to show a much larger effect on protein variance once normalized to the general power-law relationship (σ^2^∝μ^1.69^, Figure 1).

Several variables have previously been correlated with increases in noise including changes in transcription [13] and translation rates [2], [20], [21], the presence of a TATA box [2], [13], [21] and promoter positioned nucleosomes [2], [15], [16], [27]. Our model suggested that only variables involved in promoter activation should significantly increase protein variance when normalized to their mean levels, whereas variables affecting downstream processes would not.

To test this hypothesis, we correlated the protein variance residuals with variables that reflect changes in promoter activation, and with variables that affect downstream processes. Genes with TATA boxes or promoter-positioned nucleosomes, factors which influence promoter activation, had high values of residual variance (Figure 3b), indicating that they increase noise by modulating the power-law. In contrast, differences in measured rates of mRNA synthesis and degradation [28], rates of protein degradation [29], measures of ribosomal occupancy [30], and the Codon Adaptation Index [31] showed little or no correlation with residual variance (Figure 3b). This result demonstrates that these variables, which affect processes downstream of promoter activation, influence cell-to-cell protein variance almost exclusively by changing mean levels of gene expression. Taken together, the results support our hypothesis and suggest that positioned nucleosomes may account for a large portion of the residual variance.

### Promoter-positioned nucleosomes increase variance by slowing promoter activation kinetics

**Figure 4:**
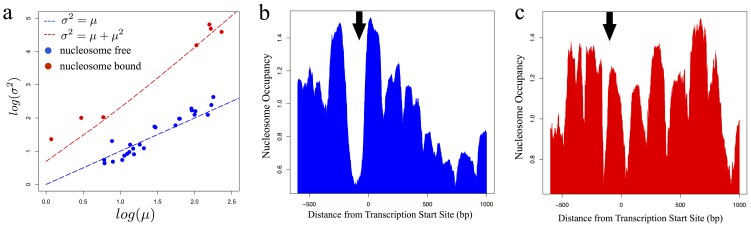
Analysis of mRNA distributions connects underlying promoter kinetics to nucleosome occupancy. a) mRNA mean and variance in *S. cerevisiae* plotted against each other in log-scale. Blue dashed line indicates the expected relationship between mean and variance in a regime of slow activation and fast inactivation rate (σ^2^ = μ), red dashed line indicates expected relationship at slow promoter kinetics (σ^2^ = μ+μ^2^). Circles represent experimental values of mRNA mean and variance (color matches best fit to promoter kinetics regime) b) Average nucleosome occupancy between −600 to +1000 relative to the TSS of *S. cerevisiae* genes exhibiting linear mRNA mean-variance scaling. The position of the canonical nucleosome free region is indicated by the black arrow. c) Same as b) but with respect to *S. cerevisiae* genes exhibiting quadratic mRNA mean-variance scaling.

Our model suggests that the increase in residual protein variance caused by positioned nucleosomes is the result of slower promoter activation in these genes. To test this hypothesis, we examined single-cell mRNA measurements performed for different genes in *S. cerevisiae* [32], since the relationship between mRNA mean and variance can be used to clearly distinguish groups of genes with different promoter kinetics [23] (see materials and methods). Our prediction is that genes without promoter-positioned nucleosomes (Figure 4b) will have fast promoter activation kinetics and thus display an approximately linear relationship between mean and variance (Figure 4a, blue line, see Supporting Information S1, section 1.8). Indeed, this was observed in the single-cell mRNA data (see Figure 4a, red dots). In contrast, our model predicts that genes with promoter-positioned nucleosomes (Figure 4c) will have slow promoter activation kinetics and will therefore display a quadratic scaling between mean and variance (Figure 4a, red line, see Supporting Information S1, section 1.8). This was again confirmed as genes lacking a nucleosome-free region displayed the predicted mean-variance relationship (Figure 4a, red dots).

### Experimental confirmation of the effects of promoter kinetics on the mean-variance relationship

**Figure 5:**
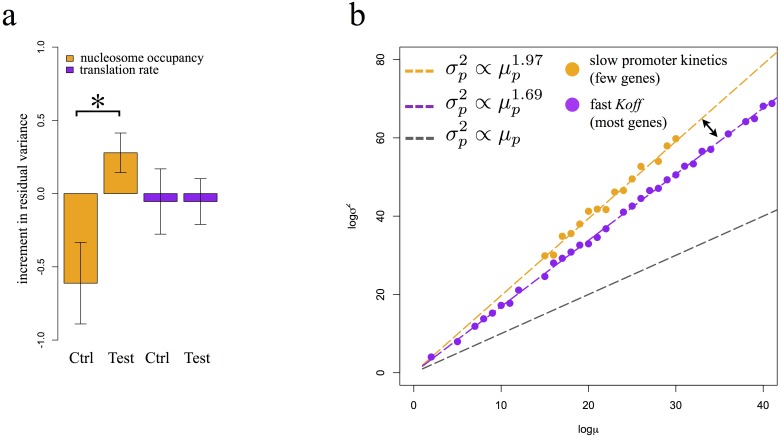
Modulating promoter kinetics changes protein mean-variance scaling. a) Increment in residual variance from glucose to ethanol in genes that show increased occupancy in ethanol (orange set: test) and genes with unaltered occupancy (orange set: control) compared to the same genes ranked by high (purple set: test) or low (purple set: test) increase in translation rate (purple set) (* indicates p<0.05, t-test). b) Diagram connecting the power-law exponent to promoter kinetics: most genes in *S. cerevisiae* exhibit promoter kinetics characterized by fast inactivation rate (purple dots) and display protein mean-variance scaling dictated by a power-law with 1.69 exponent (purple line). A small set of genes (orange dots) exhibit slow promoter kinetics and consequently present protein mean-variance scaling dictated by a quadratic scaling (orange line).

Finally, to obtain additional support for these findings, we experimentally tested whether changes in nucleosome occupancy could produce an increase in the mean-independent component of protein variance. Using *in vivo* nucleosome positioning data [33], we selected a set of *S*. *cerevisiae* TATA-containing genes whose promoters are nucleosome free in glucose but which acquire a positioned nucleosome in ethanol. A prediction of our analysis is that such genes would display increased residual variance when switched from glucose-containing medium to ethanol-containing medium. We measured the distribution of fluorescence of GFP-tagged fusion strains [29] in both glucose and ethanol by flow-cytometry, and computed the residual variance above what is expected from the mean-variance relationship. We observed a significant increase in residual variance as cells were shifted from glucose to ethanol relative to a control set of genes in which nucleosomes do not change between the two conditions (p-value <0.05, T-test across 3 biological replicates, Figure 5a nucleosome occupancy set).

Using this same gene set, we examined whether changes in protein translation rate affected the mean-independent component of the variance. Our model predicts that translation rate should not correlate with residual variance, and we did not observe any significant difference (p-value >0.4, T-test across 3 biological replicates, Figure 5a translation rate set). These results support our hypothesis that positioned nucleosomes are the major source of mean independent noise. We conclude that nucleosome bound promoters showed higher protein variance as a result of slowed promoter activation kinetics, which increases the exponent of the power-law-like relationship between protein mean and variance. These results can be summarized in a general model: most of the genes in *S. cerevisiae* exhibit promoter kinetics characterized by fast inactivation rate and as a result display a protein mean-variance scaling dictated by a general power-law relationship with exponent equal to 1.69 (Figure 5b, purple dots and line). In contrast, few genes characterized by slow promoter kinetics display approximate quadratic scaling between protein mean and variance (Figure 5b, orange dots and line). Changes in promoter kinetics induced by nucleosome positioning can affect this relationship, resulting in an increase in protein variance compared to the general power-law.

## Discussion

Single-cell variance in protein levels plays a major role in generating phenotypic differences [4], [5]. A fundamental property of protein variance is its dependence on mean protein levels through a power-law-like relationship. This relationship holds in yeast (σ^2^∝μ^1.6^), bacteria (σ^2^∝μ^1.5^) [24] and human T-cells (σ^2^∝μ^1.7^) [34], suggesting the processes that determine the power-law are common across different species.

Using a stochastic model of gene expression parameterized with empirically measured kinetic rates [13], [25], [28], we found that the power-law is a natural consequence of the kinetics of transcription and translation, fundamental mechanisms shared between these three organisms. Through the same framework, we also were able to predict for the first time protein variance at a genomic scale. Molecular processes that differ significantly between these species, such as chromatin structure, nuclear export, or unequal partitioning during the cell cycle, were not required to explain the power-law nor to predict protein variance.

These results were reached by fitting a global rate of promoter activation and assuming the same promoter kinetic regime across the whole genome. Although this is in fact an approximation as it would be unrealistic to expect all promoter to be activated at the same rate, we found this assumption to be largely true in promoter bashing experiments [14]. Furthermore, this result suggests a model where changes in promoter initiation arise mostly as a result in changes of promoter inactivation rather than activation, a result that has been empirically observed at a single gene level in different organisms [23], [35].

The global regime of promoter initiation that we captured consisted in a fast promoter inactivation rate and slow activation rates, resulting in short burst frequency (0.59 min^−1^) and an average small burst size (0.104 transcripts per burst on average). These values are in agreement with the only direct empirical measure of transcriptional initiation in *S. cerevisiae* [26]. In this kinetic regime, most promoter transitions to the active state do not produce an mRNA transcript – for the “average” gene, approximately 89% are non-productive. Transitions that do produce a transcript typically only produce a single mRNA molecule (∼9.4% of transitions, for the average of transitions, for transitions produce multiple transcripts (0.5%). In this regime, RNA production very nearly follows a Poisson process, with σ^2^∝μ^1.1^. However, this small non-linearity between mean and variance is amplified at the protein level and the mean-variance relationship follows the σ^2^∝μ^1.69^ power-law-like relationship.

One practical application of understanding the power law is that it allows to separate different mechanisms that contribute to the increase of protein variance. By using the power-law obtained under these rates, 97% of all protein variance across the genome can explained solely by mean protein levels, suggesting that this kinetic regime is a general feature of transcription in *S. cerevisiae*. The 3% of genes with excess variance (up to twenty-fold over the expected variance) is consistent with the occurrence of slow promoter kinetics, which our data suggests is caused for the most part by positioned nucleosomes on their promoters. The association of nucleosomes and chromatin related factors to increased promoter variance is not novel and it has been previously observed in several studies [2], [15], [16], [27]. However, we find that nucleosome positioning is by far the dominant factor, explaining most of the excess variance. This result is even stronger when nucleosome occupancy is analyzed in the context of TATA-containing genes, a notorious class of genes characterized by higher protein variance than the rest of the proteome [2], [13], [21]. Interestingly, a recent analysis of the effect of TATA-box using synthetic promoter libraries has revealed the TATA-box not to be sufficient to increase protein variance [14]. This suggests that perhaps an interplay between TATA and chromatin architecture is required to produce the observed increase in noise, a conclusion supported by our observation in genomic data as well as in promoter mutagenesis libraries [36]. In disagreement with previous observations [2], [13], [20], [21], factors involved in molecular processes occurring after promoter initiation do not produce an excess of variance beyond what is expected. The analysis of the model explains this observation: factors modulating the kinetics of promoter initiation will produce an increase in the exponent of the power-law for that particular gene, which will result in an apparent excess of protein variance. In contrast, factors operating downstream will produce an increase in variance solely through an increase in mean following the power-law exponent specified by the kinetics of the controlling promoter. Our work therefore suggests that the power-law is a universal feature of protein expression whose particular shape is determined by the rates at which promoters transition between their active and inactive states [37], [38].

The performance of our model and the conclusions of our analysis pertain only to the intrinsic, or gene specific [10] portion of protein variance, as the dataset that we analyzed minimized the effect of global or extrinsic factors through gating [13]. The reduced extrinsic component of this dataset may also explain the absence of association of translation specific factors to excess protein variance, as previous genetic dissection revealed their enrichment among factors modulating global variance changes [18].

Finally, we did not observe any genes with variances significantly below that expected from the power-law. Reducing protein variance may be difficult for the cell due to physical constraints that render this process energetically dis-advantageous. A theoretical analysis on the limits of suppression of molecular fluctuations [39] supports this observation. Alternatively, it is possible that cells have evolved regulatory networks with intrinsic robustness to molecular fluctuations [40], suggesting that even if achievable, noise reduction may not be necessary.

Identifying the sources of noise and their underlying mechanisms is an important step in determining their role in increasing fitness. The work presented here provides a way to isolate mean-independent effects from protein variance and to connect them to their biophysical origins. A long-standing question regarding stochastic gene expression is its role in fitness [4]. Through this framework, it will be possible to completely decouple the role of protein variance from the mean, allowing a better understanding of the functional and evolutionary constraints that shape gene expression variance.

## Methods

### Data Sources

We used single-cell protein mean and variance values from flow-cytometry measurements on *S. cerevisae* GFP-fusion strains grown in YPD for ∼2000 genes from Newman *et al.* [13]. mRNA level measurements in YPD and YPEtOH were obtained from Gasch *et al.* [41]. We acquired mRNA synthesis and degradation rates from Miller *et al.* [28]. mRNA single-cell measurement data were obtained from Gandhi *et al.* [32]. Nucleosome occupancy was assessed from mnase-seq datasets in YPD and YPEtOH from Kaplan *et al.* [33]. We used protein mean and variance from synthetic promoter libraries from the work of Mogno *et al.* [14]. Definition of TATA-containing and TATA-less were obtained from Basehoar *et al.* [42]. We obtained *in vivo* ribosome occupancy profiles for each mRNA species measured in YPD from Ingolia *et al.* [30]. Data and source code generated and used in this work can be found at http://cgs.wustl.edu/~fvallania/5_noise_2011/5_noise_website/NOISE_Project_supporting_materials.html.

### Analysis of the relationship between protein mean and variance

Using single-cell protein mean and variance values in *S. cerevisiae* [13], we assumed that the underlying relationship between mean and variance could be non-linear and exponential in nature. This formulation can be generally expressed as 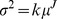
where k is a scaling factor and J is the exponential index. In log-space, this equation transforms into 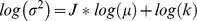
where J can now be directly calculated as the slope of a linear regression. We estimated the fraction of variance explained by the mean as the r^2^ of the regression. Variance residuals originated from this fit were defined as mean-independent variance. Regression analysis was performed using the R programming language.

### Stochastic modeling of protein and mRNA variance

To model mRNA and protein variance in *S. cerevisae*, we used analytical stochastic models derived from the solution of a system of stochastic differential equations as previously described [25]. This model describes the steady-state value of mRNA and protein variance as a function of the kinetic rates for protein activation and inactivation (*Kon* and *Koff*), mRNA synthesis and degradation (*Km* and *Dm*), and protein translation and degradation (*Kp* and *Dp*). The model for mRNA variance is expressed as 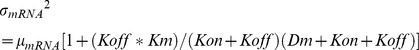
whereas for protein variance, the equation is: 
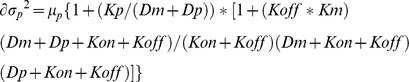



In order to predict genome-wide protein variance in *S. cerevisiae*, we assumed *Kon* and *Koff* to be uniform across the genome and fit their values. Fitting, prediction and cross-validation were computed in Perl. Analysis of the fit was performed in R. (for complete explanation see Supporting Information S1).

### Correlation analysis between mean-independent variance and molecular properties

We compared mean-independent variance to mRNA synthesis rate, mRNA degradation rate, ribosomal occupancy and CAI (Codon Adaptation Index). CAI was computed as previously described [31]. To determine the amount of variation of noise explained explained by each property, we correlated mean-independent variance with the log of the measure of each property and calculated the Pearson's correlation coefficient. We used a linear regression in log scale to avoid any non-linear effects. Regression analysis was performed in R.

### Regression model between mean-independent variance and nucleosome occupancy

We computed the Pearson's correlation coefficient between mean-independent variance and nucleosome occupancy at a single base resolution for each base ranging from −1000 to +600 relative to the transcription start site of each gene in *S. cerevisae* for which we had both nucleosome data and residual mean-independent variance. For each base, we obtained a correlation value, which was plotted as a function of its position relative to the TSS. We repeated this analysis focusing on TATA-containing and TATA-less genes only. In order to estimate the amount of variation explained by nucleosome occupancy on TATA-containing genes, we applied a linear model to predict residual mean-independent variance as a function of nucleosome occupancy. We performed a forward-regression strategy to determine the positions in the promoter sequence to be used as predictive features for our model followed by leave-one-out cross-validation to assess over-fitting (Supporting Information S1 for details). Regression analysis was performed in R.

### Experimental measurement of mean-independent variance as a function of nucleosome occupancy

We selected 15 yeast genes that acquired a nucleosome when grown in YPEtOH compared to YPD using genome-wide nucleosome occupancy data [33] (YAL054C, YBL015W, YBL075C, YBR139W, YBR145W, YDL097C, YER081W, YFL021W, YGL040C, YGL197W, YLR042C, YMR315W, YNL241C, YOL143C, YOR084W, YPR127W). We constructed a second set (control set) of 15 genes either stable nucleosome-bound or nucleosome-free promoters (YBR066C, YBR092C, YER056C-A, YJL200C, YKL071W, YLR177W, YNL112W, YOR355W, YAL060W, YDR055W, YDR495C, YDR533C, YDL222C, YER054C). For each gene in each set, we grew a corresponding GFP-fusion *S. cerevisiae* strain [29] in YPD and YPEtOH to log phase and measured single-cell protein levels using a Beckmann-Coulter Cytomics FC500 MPL flow-cytometer (Beckmann Coulter, Fullerton, CA) as previously described previously [13]. We calculated residual variance from mean and variance as described above and, for each gene we computed differential residual mean independent variance between YPEtOH and YPD. We then tested for increase in residual variance between the test and control set using one-sided t-test. Additionally, we computed the translation rate for each gene in both conditions (described in Supporting Information S1, section 1.2) and computed the differential translation rate (Δ*Kp*) between conditions (defined as *Kp*_YEtOH_ - *Kp*_YPD_). We then ranked the genes by decreasing Δ*Kp* and tested for increase in residual variance between the top and bottom half of this set using one-sided t-test. Statistics were performed in R.

## Supporting Information

Figure S1 **Changes in gene expressions are driven by changes in** ***K_off_*** **or** ***K_m_*** **whereas** ***K_on_*** **remains largely constant.** (a) Expected relationship of the VMR (upper half, blue line) and the CV (lower half, red line) with protein mean levels (µ_p_) assuming constant *K_off_* and *K_m_* and variable *K_on_*. (b) Same as in (a) but assuming instead constant *K_on_* and variable *K_off_* or *K_m_*. Equations indicate the slope of the line for the VMR-mean relationship (upper half) and the equation of the asymptotic line for the CV-mean relationship. (c) Experimentally observed relationship of the VMR and CV with protein mean levels in a promoter library dataset (Mogno et al. 2010).
(TIFF)

Figure S2 **Distinguishing between fast kinetics and short initiation events promoter regimens.** (a) Protein mean-variance relationships in promoter bashing/induction experiments: the regimes of fast promoter kinetics and short initiation events produce a linear and super linear relationship between protein mean and variance respectively. (b) Illustration of promoter activation regimens dictated by fast promoter kinetics, short initiation events, and slow bursty kinetics. In each plot, the x-axis indicates time and the y-axis indicates promoter activity. Purple points and bars represent short or extended period of promoter activation. In the case of fast promoter kinetics, the transition between active and inactive is so rapid that the activation is approximated as constant. (c) Protein mean-variance relationship in a synthetic promoter library dataset (Mogno et al 2010) in log-log plot.
(TIFF)

Figure S3 **Description and results of the experimental validation.** (a) Experimental de- sign: We selected 15 genes that acquired a nucleosome when grown in YPEtOH compared to YPD using genome-wide nucleosome occupancy data. A control set of equal size was also built with genes with stable nucleosomes across the two conditions. For each gene in each set, we grew a corresponding GFP-fusion *S. cerevisiae* strain in YPD and YPEtOH to log phase and measured single-cell protein levels by flow-cytometry. (b) Representative results of 3 yeast strains from the test group. For each strain, the distribution of fluorescence intensity is shown in YPD (cyan) and YPEtOH (purple) respectively. The amount of residual variance (labeled as MIV or mean-independent variance) is displayed under each histogram. (c) Same as in (b) but for representative strains from the control group.
(TIFF)

Table S1 **List of parameters used in the stochastic model and their source.**
(TIFF)

Supporting Information S1 **Supplementary methods, calculations, and derivations for the equations used in the main manuscript.**
(PDF)

## References

[1] Kaern M, Elston TC, Blake WJ, Collins JJ (2005) Stochasticity in gene expression: from theories to phenotypes. Nat Rev Genet 6: 451–464 10.1038/nrg1615 15883588

[2] Raser JM (2004) Control of Stochasticity in Eukaryotic Gene Expression. Science 304: 1811–1814 10.1126/science.1098641 15166317 PMC1410811

[3] Raj A, van Oudenaarden A (2008) Nature, Nurture, or Chance: Stochastic Gene Expression and Its Consequences. Cell 135: 216–226 10.1016/j.cell.2008.09.050 18957198 PMC3118044

[4] Blake WJ, Balázsi G, Kohanski MA, Isaacs FJ, Murphy KF, et al (2006) Phenotypic Consequences of Promoter-Mediated Transcriptional Noise. Mol Cell 24: 853–865 10.1016/j.molcel.2006.11.003 17189188

[5] Beaumont HJE, Gallie J, Kost C, Ferguson GC, Rainey PB (2009) Experimental evolution of bet hedging. Nature 462: 90–93 10.1038/nature08504 19890329

[6] Balaban NQ (2004) Bacterial Persistence as a Phenotypic Switch. Science 305: 1622–1625 10.1126/science.1099390 15308767

[7] Wernet MF, Mazzoni EO, Ccedil Elik A, Duncan DM, Duncan I, et al (2006) Stochastic spineless expression creates the retinal mosaic for colour vision. Nature 440: 174 10.1038/nature04615 16525464 PMC3826883

[8] Chang HH, Hemberg M, Barahona M, Ingber DE, Huang S (2008) Transcriptome-wide noise controls lineage choice in mammalian progenitor cells. Nature 453: 544–547 10.1038/nature06965 18497826 PMC5546414

[9] Raj A, Rifkin SA, Andersen E, van Oudenaarden A (2010) Variability in gene expression underlies incomplete penetrance. Nature 463: 913–918 10.1038/nature08781 20164922 PMC2836165

[10] Elowitz MB (2002) Stochastic Gene Expression in a Single Cell. Science 297: 1183–1186 10.1126/science.1070919 12183631

[11] Stewart-Ornstein J, Weissman JS, El-Samad H (2012) Cellular Noise Regulons Underlie Fluctuations in Saccharomyces cerevisiae. Mol Cell 45: 483–493 10.1016/j.molcel.2011.11.035 22365828 PMC3327736

[12] Ansel J, Bottin H, Rodriguez-Beltran C, Damon C, Nagarajan M, et al (2008) Cell-to-Cell Stochastic Variation in Gene Expression Is a Complex Genetic Trait. PLoS Genet 4: e1000049 10.1371/journal.pgen.1000049.s006 18404214 PMC2289839

[13] Newman JRS, Ghaemmaghami S, Ihmels J, Breslow DK, Noble M, et al (2006) Single-cell proteomic analysis of S. cerevisiae reveals the architecture of biological noise. Nature 441: 840–846 10.1038/nature04785 16699522

[14] Mogno I, Vallania F, Mitra RD, Cohen BA (2010) TATA is a modular component of synthetic promoters. Genome Res 20: 1391–1397 10.1101/gr.106732.110 20627890 PMC2945188

[15] Tirosh I, Sigal N, Barkai N (2010) Divergence of nucleosome positioning between two closely related yeast species: genetic basis and functional consequences. Molecular Systems Biology 6 . doi:10.1038/msb.2010.20. 10.1038/msb.2010.20 PMC2890324 20461072

[16] Choi JK, Kim YJ (2009) Intrinsic variability of gene expression encoded in nucleosome positioning sequences. Nat Genet 41: 498–503 10.1038/ng.319 19252489

[17] Bai L, Charvin G, Siggia ED, Cross FR (2010) Nucleosome-Depleted Regions in Cell-Cycle-Regulated Promoters Ensure Reliable Gene Expression in Every Cell Cycle. Dev Cell 18: 544–555 10.1016/j.devcel.2010.02.007 20412770 PMC2867244

[18] Rinott R, Rinott R, Jaimovich A, Jaimovich A, Friedman N, et al (2011) Exploring transcription regulation through cell-to-cell variability. Proc Natl Acad Sci USA 108: 6329–6334 10.1073/pnas.1013148108 21444810 PMC3076844

[19] Bar-Even A, Paulsson J, Maheshri N, Carmi M, O'Shea E, et al (2006) Noise in protein expression scales with natural protein abundance. Nat Genet 38: 636–643 10.1038/ng1807 16715097

[20] Ozbudak EM, Thattai M, Kurtser I, Grossman AD, van Oudenaarden A (2002) Regulation of noise in the expression of a single gene. Nat Genet 31: 69–73 10.1038/ng869 11967532

[21] Blake WJ, Blake WJ, Kaern M, Kaern M, Cantor CR, et al (2003) Noise in eukaryotic gene expression. Nature 422: 633–637 10.1038/nature01546 12687005

[22] Huh D, Paulsson J (2010) Non-genetic heterogeneity from stochastic partitioning at cell division. Nat Genet 43: 95–100 10.1038/ng.729 21186354 PMC3208402

[23] So LH, Ghosh A, Zong C, Sepúlveda LA, Segev R, et al (2011) General properties of transcriptional time series in Escherichia coli. Nat Genet 43: 554–560 10.1038/ng.821 21532574 PMC3102781

[24] Taniguchi Y, Choi PJ, Li GW, Chen H, Babu M, et al (2010) Quantifying E. coli Proteome and Transcriptome with Single-Molecule Sensitivity in Single Cells. Science 329: 533–538 10.1126/science.1188308 20671182 PMC2922915

[25] Paulsson J (2005) Models of stochastic gene expression. Physics of Life Reviews 2: 157–175.

[26] Larson DR, Zenklusen D, Wu B, Chao JA, Singer RH (2011) Real-Time Observation of Transcription Initiation and Elongation on an Endogenous Yeast Gene. Science 332: 475–478 10.1126/science.1202142 21512033 PMC3152976

[27] Weinberger L, Voichek Y, Tirosh I, Hornung G, Amit I, et al. (2012) Expression Noise and Acetylation Profiles Distinguish HDAC Functions. Mol Cell. doi:10.1016/j.molcel.2012.05.008. 10.1016/j.molcel.2012.05.008 PMC3408861 22683268

[28] Miller C, Schwalb B, Maier K, Schulz D, Dümcke S, et al (2011) Dynamic transcriptome analysis measures rates of mRNA synthesis and decay in yeast. Molecular Systems Biology 7: 458–458 10.1038/msb.2010.112 21206491 PMC3049410

[29] Ghaemmaghami S, Ghaemmaghami S, Huh WK, Huh WK, Bower K, et al (2003) Global analysis of protein expression in yeast. Nature 425: 737–741 10.1038/nature02046 14562106

[30] Ingolia NT, Ghaemmaghami S, Newman JRS, Weissman JS (2009) Genome-wide analysis in vivo of translation with nucleotide resolution using ribosome profiling. Science 324: 218–223 10.1126/science.1168978 19213877 PMC2746483

[31] Sharp PM, Li WH (1987) The codon Adaptation Index–a measure of directional synonymous codon usage bias, and its potential applications. Nucleic Acids Research 15: 1281–1295. 3547335 10.1093/nar/15.3.1281 PMC340524

[32] Gandhi SJ, Gandhi SJ, Zenklusen D, Zenklusen D, Lionnet T, et al (2011) Transcription of functionally related constitutive genes is not coordinated. Nat Struct Mol Biol 18: 27–34 10.1038/nsmb.1934 21131977 PMC3058351

[33] Kaplan N, Moore IK, Fondufe-Mittendorf Y, Gossett AJ, Tillo D, et al (2008) The DNA-encoded nucleosome organization of a eukaryotic genome. Nature 458: 362–366 10.1038/nature07667 19092803 PMC2658732

[34] Skupsky R, Burnett JC, Foley JE, Schaffer DV, Arkin AP (2010) HIV Promoter Integration Site Primarily Modulates Transcriptional Burst Size Rather Than Frequency. PLoS Comp Biol 6: e1000952 10.1371/journal.pcbi.1000952.s007 PMC2947985 20941390

[35] Suter DM, Molina N, Gatfield D, Schneider K, Schibler U, et al (2011) Mammalian genes are transcribed with widely different bursting kinetics. Science 332: 472–474 10.1126/science.1198817 21415320

[36] Hornung G, Hornung G, Oren M, Oren M, Barkai N, et al (2012) Nucleosome Organization Affects the Sensitivity of Gene Expression to Promoter Mutations. Mol Cell 46: 362–368 10.1016/j.molcel.2012.02.019 22464732 PMC3356688

[37] Carey LB, van Dijk D, Sloot PMA, Kaandorp JA, Segal E (2013) Promoter sequence determines the relationship between expression level and noise. PLoS Biol 11: e1001528 10.1371/journal.pbio.1001528 23565060 PMC3614515

[38] Sanchez A, Golding I (2013) Genetic determinants and cellular constraints in noisy gene expression. Science 342: 1188–1193 10.1126/science.1242975 24311680 PMC4045091

[39] Lestas I, Vinnicombe G, Paulsson J (2010) Fundamental limits on the suppression of molecular fluctuations. Nature 467: 174–178 10.1038/nature09333 20829788 PMC2996232

[40] Kaneko K, Kaneko K (2007) Evolution of Robustness to Noise and Mutation in Gene Expression Dynamics. PLoS ONE 2: e434 10.1371/journal.pone.0000434 17502916 PMC1855988

[41] Gasch AP, Gasch AP, Spellman PT, Spellman PT, Kao CM, et al (2000) Genomic expression programs in the response of yeast cells to environmental changes. Mol Biol Cell 11: 4241–4257. 11102521 10.1091/mbc.11.12.4241 PMC15070

[42] Basehoar AD, Basehoar AD, Zanton SJ, Zanton SJ, Pugh BF, et al (2004) Identification and distinct regulation of yeast TATA box-containing genes. Cell 116: 699–709. 15006352 10.1016/s0092-8674(04)00205-3

