# Supplementary figures and images for: Origin and Consequences of the Relationship between Protein Mean and Variance
Source: PLoS One. 2014 Jul 25;9(7):e102202. doi: 10.1371/journal.pone.0102202 (PMC4111490; doi:10.1371/journal.pone.0102202)

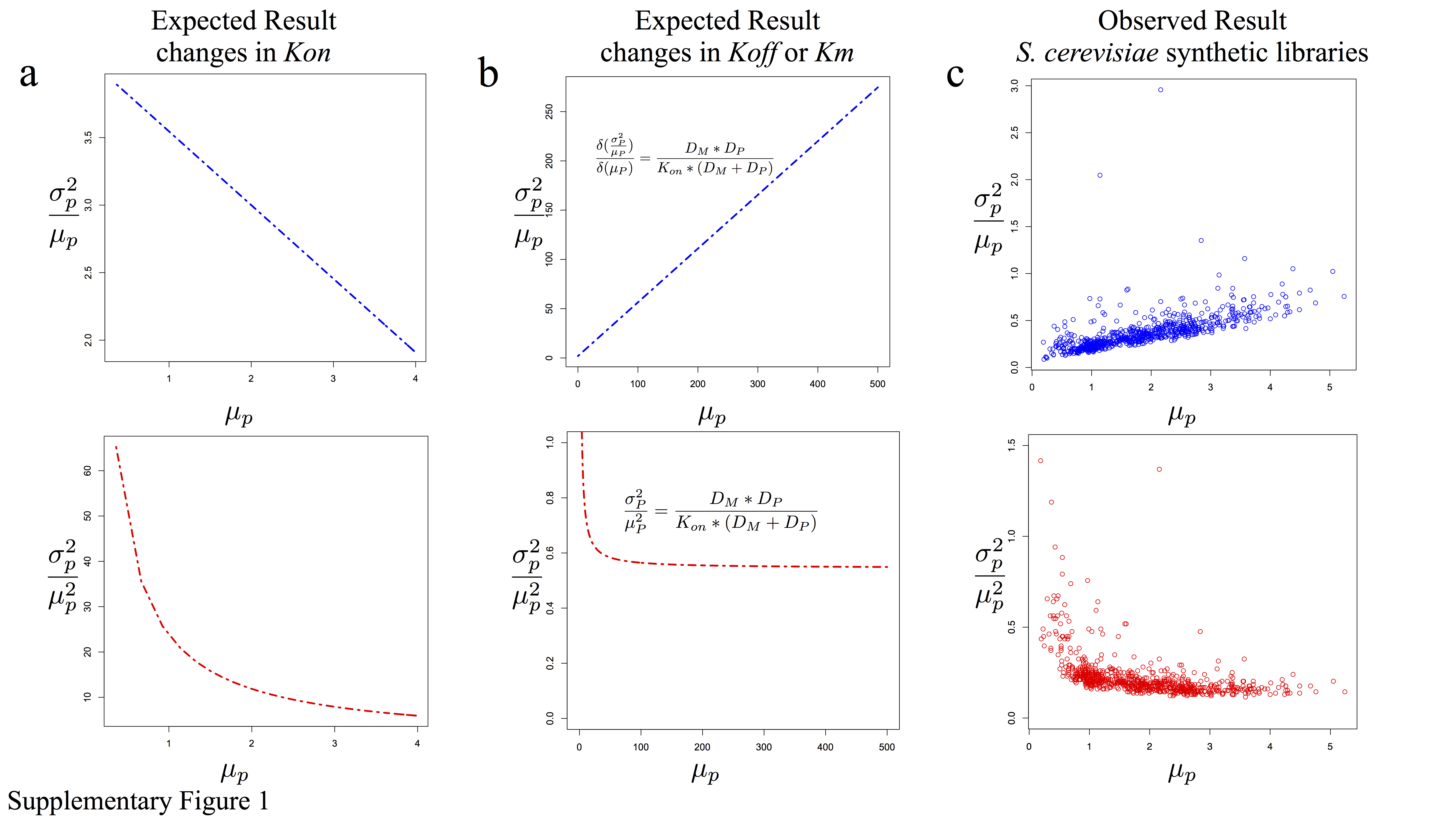

Supplement: Figure S1 — Changes in gene expressions are driven by changes in Koff or Km whereas Kon remains largely constant. (a) Expected relationship of the VMR (upper half, blue line) and the CV (lower half, red line) with protein mean levels (µp) assuming constant Koff and Km and variable Kon. (b) Same as in (a) but assuming instead constant Kon and variable Koff or Km. Equations indicate the slope of the line for the VMR-mean relationship (upper half) and the equation of the asymptotic line for the CV-mean relationship. (c) Experimentally observed relationship of the VMR and CV with protein mean levels in a promoter library dataset (Mogno et al. 2010). (TIFF) [file pone.0102202.s001.tiff]

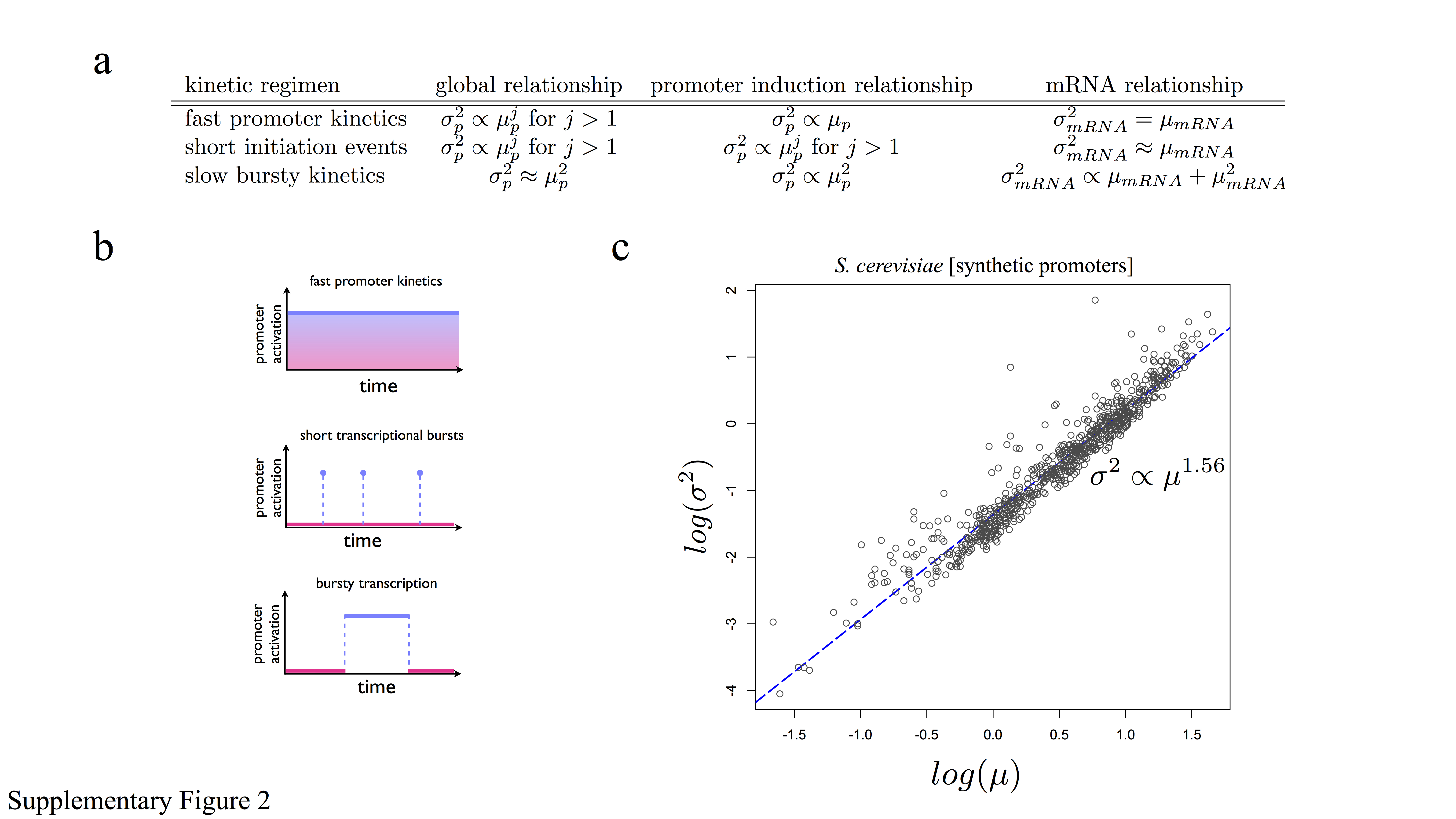

Supplement: Figure S2 — Distinguishing between fast kinetics and short initiation events promoter regimens. (a) Protein mean-variance relationships in promoter bashing/induction experiments: the regimes of fast promoter kinetics and short initiation events produce a linear and super linear relationship between protein mean and variance respectively. (b) Illustration of promoter activation regimens dictated by fast promoter kinetics, short initiation events, and slow bursty kinetics. In each plot, the x-axis indicates time and the y-axis indicates promoter activity. Purple points and bars represent short or extended period of promoter activation. In the case of fast promoter kinetics, the transition between active and inactive is so rapid that the activation is approximated as constant. (c) Protein mean-variance relationship in a synthetic promoter library dataset (Mogno et al 2010) in log-log plot. (TIFF) [file pone.0102202.s002.tiff]

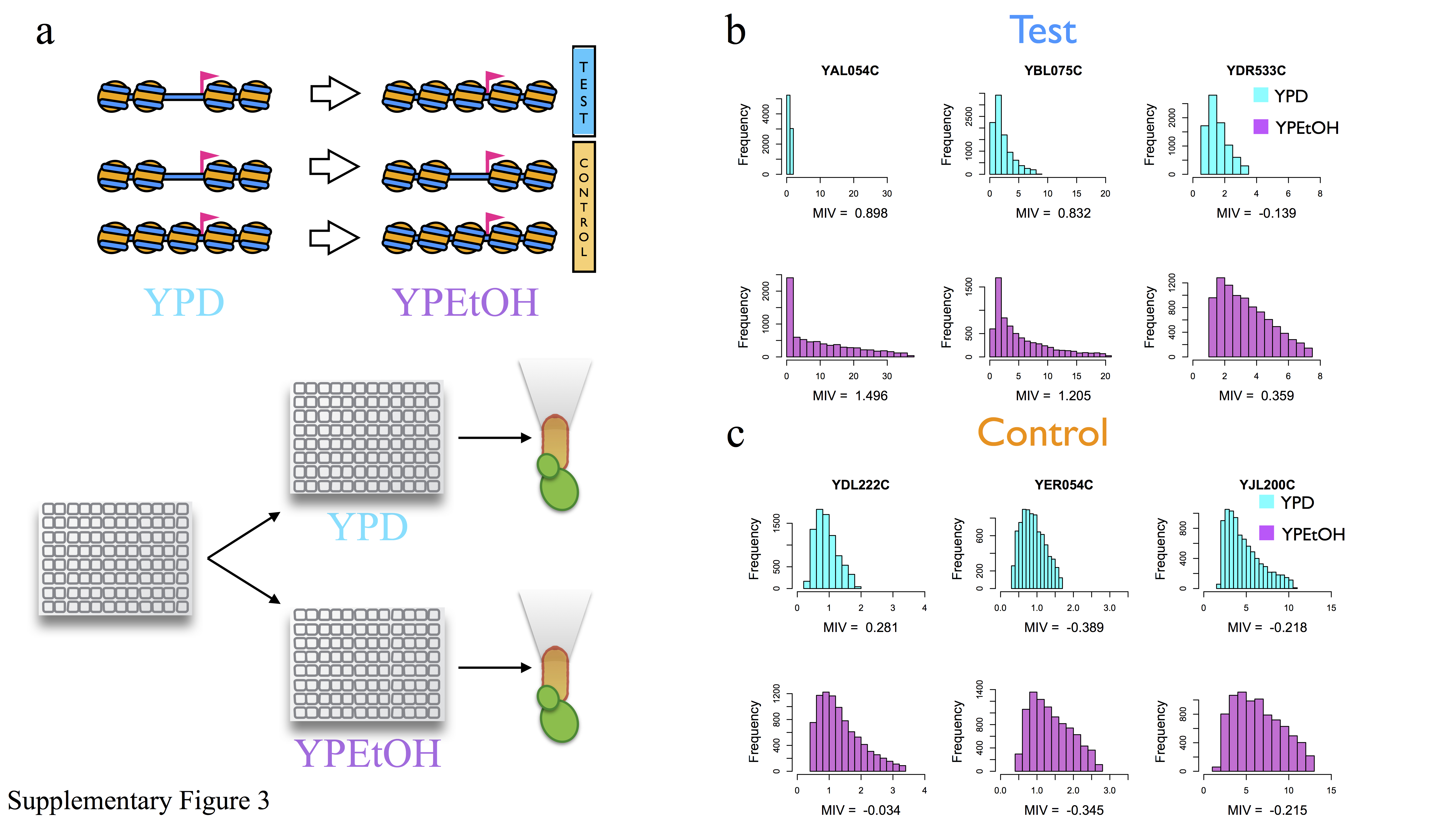

Supplement: Figure S3 — Description and results of the experimental validation. (a) Experimental de- sign: We selected 15 genes that acquired a nucleosome when grown in YPEtOH compared to YPD using genome-wide nucleosome occupancy data. A control set of equal size was also built with genes with stable nucleosomes across the two conditions. For each gene in each set, we grew a corresponding GFP-fusion S. cerevisiae strain in YPD and YPEtOH to log phase and measured single-cell protein levels by flow-cytometry. (b) Representative results of 3 yeast strains from the test group. For each strain, the distribution of fluorescence intensity is shown in YPD (cyan) and YPEtOH (purple) respectively. The amount of residual variance (labeled as MIV or mean-independent variance) is displayed under each histogram. (c) Same as in (b) but for representative strains from the control group. (TIFF) [file pone.0102202.s003.tiff]

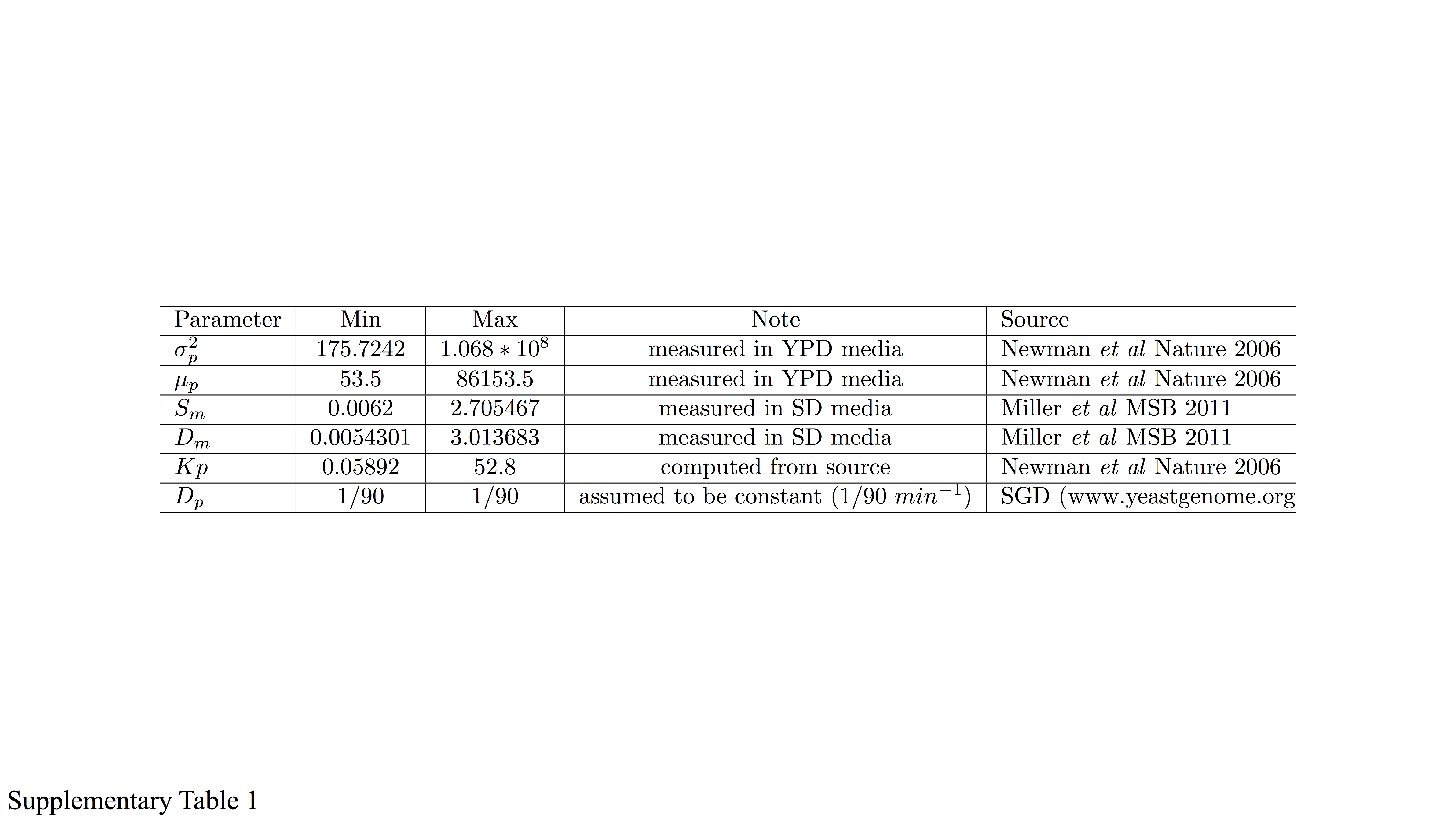

Supplement: Table S1 — List of parameters used in the stochastic model and their source. (TIFF) [file pone.0102202.s004.tiff]
