# Supplementary material for: Origin and Consequences of the Relationship between Protein Mean and Variance
Source: PLoS One. 2014 Jul 25;9(7):e102202. doi: 10.1371/journal.pone.0102202 (PMC4111490; doi:10.1371/journal.pone.0102202)
Supplement: Supporting Information S1 — Supplementary methods, calculations, and derivations for the equations used in the main manuscript. (PDF) [file pone.0102202.s005.pdf]

Supporting Information

Contents

1 Stochastic Modeling of mRNA and Protein Variance in *S.cerevisiae* **2**

1.1 Description of the Model 2

1.2 Description of input datasets and calculation of K_p 3

1.3 Fitting K_{on} and K_{off} across the entire *S cerevisiae* genome 4

1.4 Validating the assumption of a global invariant activation rate across the *S. cerevisiae* genome 5

1.5 Power-law relationship analysis 5

1.6 Power-law relationship under slow promoter kinetics 6

1.7 Power-law relationship: Randomization analysis of molecular parameters 6

1.8 Derivation of stochastic models for σ^2_{mRNA} 7

1.9 Verification the promoter kinetic regime characterized by short and infrequent initiation events 8

2 Analysis of residual protein variance **10**

2.1 Linear regression model between mean-independent variance and nucleosome occupancy . . . 10

3 Supplementary figure legends **11**

4 Supplementary references **12**

1 Stochastic Modeling of mRNA and Protein Variance in *S.cerevisiae*

1.1 Description of the Model

We used a stochastic model describing the expression of a single gene though the process of its promoter activation, inactivation, transcription at the active promoter state, degradation of the transcribed mRNA, translation of the transcribed mRNA, and degradation of the protein. A visualization of the model is described in Figure 2A. Each step is associated with a rate constant:

- K_{on} = promoter activation rate
- K_{off} = promoter inactivation rate
- K_m = mRNA transcription rate of active promoter
- D_m = mRNA degradation rate
- K_p = mRNA translation rate
- D_p = protein degradation rate

The model that we used is the analytical solution of a chemical master equation describing σ^2 for mRNA and protein at steady-state. This solution and its derivation have been previously described (Paulsson , 2005). The value of σ^2 for mRNA levels at steady-state corresponds to:

$$\sigma^2_{mRNA} = \mu_{mRNA}[1 + \frac{K_{off}K_m}{(K_{on} + K_{off})(D_m + K_{on} + K_{off})}] \tag{1}$$

whereas σ^2 for protein levels at steady-state is defined as:

$$\sigma^2_p = \mu_p\{1 + \frac{K_p}{D_m + D_p}[1 + \frac{(K_{off}K_m)(D_m + D_p + K_{on} + K_{off})}{(K_{on} + K_{off})(D_m + K_{on} + K_{off})(D_p + K_{on} + K_{off})}]\} \tag{2}$$

μ_{mRNA} and μ_p are the mean mRNA and protein levels respectively and are defined as

$$\mu_{mRNA} = \frac{< P_{on} > K_m}{D_m} \tag{3}$$

$$\mu_p = \frac{< P_{on} > K_m K_p}{D_m D_p} \tag{4}$$

where $< P_{on} >$ is the probability of the promoter being active, which is specified by K_{on} and K_{off} as

$$< P_{on} >= \frac{K_{on}}{K_{on} + K_{off}} \tag{5}$$

1.2 Description of input datasets and calculation of K_p

We applied the model to a single-cell protein measurement dataset in *S.cerevisiae* (Newman et al, 2006) in order to predict σ_p^2. The dataset provides values of σ_p^2 and μ_p for ~2200 genes. Our experimentally derived parameters were obtained from the following sources:

Parameter	Source
S_m	Miller *et al* MSB 2011
D_m	Miller *et al* MSB 2011
Kp	Computed from Newman *et al* Nature 2006
D_p	assumed to be constant (1/90 min^{-1})

A more detailed description of the range of the parameters can be found in Table S1. All parameters were measured or computed from yeast strains grown in YPD with the exception of S_m and D_m, which were grown in SD medium (Miller *et al* MSB 2011). We used these two parameters under the assumption that very few difference would exist between YPD and SD conditions, making the set compatible for our purpose.

S_m is defined as the product of K_m with the probability of the promoter being active, which is specified by K_{on} and K_{off} as

$$S_m =< P_{on} > K_m \tag{6}$$

and, unlike K_m, can be experimentally measured. Because all genes were tagged with GFP, we assumed the fusion proteins to be stable and degraded at a rate equivalent to the dilution rate, i.e. 1/90 min^{-1} (reported in `http://www.yeastgenome.org/`). This value represents the lower limit of protein degradation as active degradation events will result in higher observed degradation rates. Although K_p was not readily available at a genome-wide scale, it can be easily computed from μ_p, μ_{mRNA}, and D_p as :

$$K_p = \frac{\mu_p D_p}{\mu_{mRNA}} \tag{7}$$

1.3 Fitting K_{on} and K_{off} across the entire *S cerevisiae* genome

By applying (6) to (2), we removed K_m from the model by replacing it with S_m resulting in :

$$\sigma_p^2 = \mu_p\{1 + \frac{K_p}{D_m + D_p}[1 + \frac{S_m}{K_{on}}\frac{K_{off}(D_m + D_p + K_{on} + K_{off})}{(D_m + K_{on} + K_{off})(D_p + K_{on} + K_{off})}]\} \tag{8}$$

which can be parameterized using the data described above. We estimated K_{on} and K_{off} on Newman's dataset using a gradient descent non-linear fit strategy implemented in Perl (this strategy is described in detail in Vallania et al, 2010). We obtained a value of $K_{on} = 0.59\ min^{-1}$ whereas K_{off} always converged to the maximum allowed value, therefore becoming negligible. This result indicates a regime of slow promoter activation (K_{on}) followed by fast inactivation (K_{off}). We derived an expression for protein variance under this regimen.

Starting from (8), and by assuming that K_{off} dominates over the other parameters, as determined by the fit, the equation simplifies to

$$\sigma_p^2 \approx \mu_p\{1 + \frac{K_p}{D_m + D_p}[1 + \frac{S_m}{K_{on}}]\} \tag{9}$$

where $\frac{S_m}{K_{on}}$ is equivalent to the burst size (Skupsky R *et.al.* 2010) under this regimen. This can be shown as:

$$\frac{S_m}{K_{on}} = \frac{K_m}{K_{on} + K_{off}} \approx \frac{K_m}{K_{off}} \tag{10}$$

We applied (9) to estimate σ_p^2 in *S.cerevisiae* using the estimated value of K_{on} and empirically measured values for the remaining parameters. We obtained a real scale correlation of r = 0.839 and a log scale correlation of r = 0.962 between predicted and measured σ_p^2. The results of this prediction are plotted in Figure 2b.

To check for over-fitting, we performed cross-validation by randomly sampling 50% of the dataset as training set and the second 50% as a validation set 100 times. For each round, we estimated K_{on} and K_{off} from the training set and evaluated the goodness of fit on the validation set ($r = 0.957 \pm 0.018$, $p < 2.2 * 10^{-16}$).

1.4 Validating the assumption of a global invariant activation rate across the *S. cerevisiae* genome

The results of our fit indicated a fast inactivation rate and suggested a slow and general value of K_on across the $S.cerevisiae$ genome. Previous experiments (Skupsky R $et.al.$ 2010) suggest that in mammalian cells it is the case, as mean and variance are affected by changes in burst size ($\frac{K_m}{K_{off}}$) rather than changes in burst frequency (K_{on}).

In order to test whether this assumption applied to our system, we analyzed a promoter dataset described above (Mogno et al, 2010). In this dataset, a library of different promoters was cloned upstream the same reporter gene. As a result, K_p,D_m,and D_p can be assumed as constants in the system. We asked whether changes in promoter activity in the libraries were due to changes in burst frequency K_{on} or burst size ($\frac{K_m}{K_{off}}$). To do so, we analyzed the relationship between the VMR ($\frac{\sigma_p^2}{\mu_p}$) and CV^2 ($\frac{\sigma_p^2}{\mu_p^2}$) as a function of μ_p^2. Following the expression of σ_p^2 in (9), we define:

$$\frac{\sigma_p^2}{\mu_p} = 1 + \frac{K_p}{D_m + D_p} + \frac{K_p}{D_m + D_p}\frac{K_m}{K_{on} + K_{off}} \tag{11}$$

$$\frac{\sigma_p^2}{\mu_p^2} = \frac{1}{\mu_p} + \frac{K_p}{(D_m + D_p) * \mu_p} + \frac{(D_m + D_p)D_m D_p}{K_{on}} \tag{12}$$

where the parameters represented in blue indicate constants. Changes in K_{on} will result in a inverse relationship between both mean and VMR and mean and CV^2 (Figure S1a). In contrast, changes in burst size (by either K_m or K_{off}) with constant burst frequency would result in a linear relationship between VMR and μ_p and an asymptotically constant relationship between CV^2 and μ_p (Figure S1b). The experimental data from Mogno *et al* follows the second model which assumes changes in burst size with constant K_{on}, thereby validating our assumption (Figure S1c).

1.5 Power-law relationship analysis

To estimate the exponent of the power-law relationship generated between protein mean levels (μ_p) and predicted protein variance (σ_p^2)in the Newman et al dataset we performed log-log regression between σ_p^2 and μ_p which is defined as

$$log(\sigma_p^2) = j * log(\mu_p) + log(k) \tag{13}$$

corresponding to

$$\sigma_p^2 = k * \mu_p^j \tag{14}$$

in real space, where j is the exponent of the power-law.

1.6 Power-law relationship under slow promoter kinetics

We performed the same power-law analysis described above for a promoter regime characterized by slow activation and inactivation kinetics. Under this regimen $K_{on}, K_{off} << K_m, D_m, K_p, D_p$ and the promoter contributes to σ_p^2 following a Bernoulli/Binomial process (Paulsson 2005). As described above, in this context the variance of the active promoter (σ_{prom}^2), can be expressed as

$$\sigma_{prom}^2 =< P_{on} > (1- < P_{on} >) = \frac{K_{on}K_{off}}{(K_{on} + K_{off})^2} \tag{15}$$

which will reach it's maximum value when $< P_{on} >$ and $1- < P_{on} >$ will be equivalent. As K_{on} and K_{off} are small in this regimen, by assuming maximum promoter variance, we derived an expression of mRNA variance under bursty kinetics. As K_{on} and K_{off} are small in this regimen, we can re-write (2) as

$$\sigma_p^2 \approx \mu_p\{1 + \frac{K_p}{D_m + D_p}[1 + \frac{K_m K_{off}(D_m + D_p)}{D_m D_p (K_{on} + K_{off})}]\} \approx \mu_p + \mu_p \frac{K_p}{D_m + D_p} + \mu_p^2 \tag{16}$$

We then estimated σ_p^2 in this regime by computing its values using the same set of parameters used above and again computed the exponent of the power-law relationship between predicted σ_p^2 and μ_p using (13).

1.7 Power-law relationship: Randomization analysis of molecular parameters

We developed a Monte-Carlo simulation in which each parameter in the parameter set (K_{on}, K_{off}, K_m, K_p, D_m, D_p) is randomly selected according to the known physiological distribution and range. The physiological ranges used for this simulation were as follows:

Parameter	Minimum	Maximum	Distribution/Source
K_{on}	0.5	0.6	Uniform in log-space
K_{off}	0.003	50	Measured by JR Chubb *et al* Current Biology 2006 and DM Suter *et al* Science 2011
K_m	0.05892	12.1	Computed from Sm (measured by Miller *et al* MSB 2011)
D_m	0.0054301	3.013683	Measured by Miller *et al* MSB 2011
K_p	0.05892	52.8	Computed from Newman *et al* Nature 2006
D_p	0.000042	0.3465736	measured by Ghaemmaghami S *et al* Nature 2003

K_{on} was defined by using our fitted value of 0.59 min^{-1} and determining a small range around it. K_{off} was defined from the work of (JR Chubb *et al* Current Biology 2006) and (DM Suter *et al* Science 2011). K_m was computed from K_{on},K_{off} and S_m using (6). K_p was computed from the dataset using (7). Each round of Monte-Carlo simulation randomly produced 2400 parameter sets (as large as the dataset from Newman *etal.*). For each set, a protein mean and variance were computed using the previous equations. A linear regression between the log values of mean and variance for the whole dataset was then computed. We repeated this procedure 1,000 times and finally we computed the average slope resulting from each linear regression.

1.8 Derivation of stochastic models for σ^2_{mRNA}

We derived expressions for mRNA variance under different promoter kinetics regimens. We started by expressing equation (1) as a function of S_m, resulting in

$$\sigma^2_{mRNA} = \mu_{mRNA}[1 + S_m \frac{K_{off}}{K_{on}(D_m + K_{on} + K_{off})}] \tag{17}$$

Under a regimen of fast K_{off} and slowK_{on}, this equation converges to

$$\sigma^2_{mRNA} \approx \mu_{mRNA}(1 + \frac{S_m}{K_{on}}) \tag{18}$$

We found that in our dataset, the average burst size $\frac{S_m}{K_{on}}$ corresponds to 0.1042561.

$$\sigma^2_{mRNA} \approx \mu_{mRNA} \tag{19}$$

Under a regimen of slow promoter kinetics, we expressed equation (1) as described above for (16), resulting

in:

$$\sigma^2_{mRNA} \approx \mu_{mRNA}[1 + \frac{K_{off}K_m}{(K_{on}+K_{off})D_m}] = \mu_{mRNA}[1 + (1- < P_{on} >)\frac{K_m}{D_m}] \tag{20}$$

if we assume that the promoter variance term is at its maximum, implying that $< P_{on} >$ is equal to $(1- < P_{on} >)$, then the expression simplifies to

$$\sigma^2_{mRNA} \approx \mu_{mRNA} + \mu^2_{mRNA} \tag{21}$$

1.9 Verification the promoter kinetic regime characterized by short and infrequent initiation events

The results of our fit indicate the presence of a promoter activation regimen characterized by short and infrequent initiation events. In order to verify the accuracy of our result, we compared this regimen with two alternative regimens: a regimen characterized by fast promoter kinetics (fast K_{off} and K_{on}) and a regimen with slow bursty kinetics (as described above).

Under fast promoter kinetics, K_{off} and K_{on} are assumed to be faster than any other parameter, reducing (1) and (8) to

$$\sigma^2_{mRNA} \approx \mu_{mRNA} \tag{22}$$

and

$$\sigma^2_p \approx \mu_p\{1 + \frac{K_p}{D_m + D_p}\} \tag{23}$$

respectively.

To better distinguish between the three regimens, we considered an experimental scenario where the only varying parameter was the level of promoter activation. This can be achieved by either inducing a single promoter-gene construct at multiple levels, or by generating and measuring the expression of a promoter library driving the expression of the same reporter gene. Under these experimental conditions, because only K_{off} and K_{on} would be changing, we would expect to observe

$$\sigma^2_p = \mu_p * \alpha \tag{24}$$

for fast promoter kinetics,

$$\sigma_p^2 = \mu_p * \alpha + \frac{\mu_p * (\alpha - 1)}{K_{on} + K_{off}} \tag{25}$$

for short initiation events, and

$$\sigma_p^2 = \mu_p * \alpha + \mu_p^2 \tag{26}$$

for slow bursty kinetics. α is a constant term and is equivalent to $1 + \frac{K_p}{D_m + D_p}$. As α is always greater than 1 for cases were K_p is not negligible compared to the degradation rates D_m and D_p, which we can assume if the gene is indeed expressed at detectable levels, then the underlying relationships between variance and mean are expected to be non-linear except for the case of fast promoter kinetics. Given our previous derivations and results (see above and Figure 3a), we summarized our expectations in the following table (also displayed as Figure S2a):

kinetic regimen	global relationship	promoter induction relationship	mRNA relationship
fast promoter kinetics	$\sigma_p^2 \propto \mu_p^j$ for $j > 1$	$\sigma_p^2 \propto \mu_p$	$\sigma_{mRNA}^2 = \mu_{mRNA}$
short initiation events	$\sigma_p^2 \propto \mu_p^j$ for $j > 1$	$\sigma_p^2 \propto \mu_p^j$ for $j > 1$	$\sigma_{mRNA}^2 \approx \mu_{mRNA}$
slow bursty kinetics	$\sigma_p^2 \approx \mu_p^2$	$\sigma_p^2 \propto \mu_p^2$	$\sigma_{mRNA}^2 \propto \mu_{mRNA} + \mu_{mRNA}^2$

Because 97% of the protein variance is captured by a non-linear power-law with exponent of 1.69, we can then excluded the underlying promoter kinetics to be slow bursty, as we would expect an exponent close to 2 (1.97 , see Figure 3a). To distinguish between the remaining models, we analyzed a synthetic promoter library in yeast (Mogno *et al* 2010) and plotted protein variance and mean in log scale. We observed a non-linear relationship with exponent 1.56, suggesting that the underlying relationship is indeed dictated by short initiation events.

2 Analysis of residual protein variance

2.1 Linear regression model between mean-independent variance and nucleosome occupancy

To determine the amount of variation in mean-independent variance explained by nucleosome occupancy at the promoter sequence, we built a linear model defined as

$$\hat{y} = \sum_{i}^{n} N_i * \alpha_i \tag{27}$$

where y represents the residual variance, N_i represents the value of nucleosome occupancy of the ith descriptive position used in the model, where n is the number of single-base positions in the promoter sequence that are used as input features for the model. We chose a linear model because of its simplicity and because we lacked detailed information on the promoter architecture of each analyzed gene, making a physically motivated sequence based model difficult to apply. Because the number of potential features (positions in the promoter sequence) is large, we selected the number of features to be used by the model by applying a forward regression strategy (for details see Hastie, Tibshirani, Friedman. The Elements of Statistical Learning. *Springer Books*). For each subset of features incorporated in the model, we computed a set of Monte Carlo simulations where we randomized nucleosome positions and residual variance pairing 100 times. We computed the average r^2 of the Monte Carlo simulations and compared it to the real r^2 of the model using the same set of features. We chose the set of features that maximized the increment of r^2 of the real set from the average Monte Carlo simulations. Our final set includes 149 features, namely positions in the promoter sequence with associated values of nucleosome occupancy across the dataset. To test for over-fitting, we performed a leave-one-out cross-validation strategy, where we predicted the results of 1 data-point after training the parameters on the rest of the dataset. This procedure was repeated for each data point and overall correlation was then computed. This strategy was implemented in the R programming language.

Datasets, source code and results can be accessed and downloaded at `http://cgs.wustl.edu/~fvallania/5_noise_2011/5_noise_website/NOISE_Project_supporting_materials.html`

3 Supplementary figure legends

Figure S1: Changes in gene expressions are driven by changes in K_{off} or K_m whereas K_{on} remains largely constant. (a) Expected relationship of the VMR ($\frac{\sigma_p^2}{\mu_p}$) (upper half, blue line) and the CV ($\frac{\sigma_p^2}{\mu_p^2}$) (lower half, red line) with protein mean levels (μ_p) assuming constant K_{off} and K_m and variable K_{on}. (b) Same as in (a) but assuming instead constant K_{on} and variable K_{off} or K_m. Equations indicate the slope of the line for the VMR-mean relationship (upper half) and the equation of the asymptotic line for the CV-mean relationship. (c) Experimentally observed relationship of the VMR and CV with protein mean levels in a promoter library dataset (Mogno *et al.* 2010).

Figure S2: Distinguishing between fast kinetics and short initiation events promoter regimens: (a) Protein mean-variance relationships in promoter bashing/induction experiments: the regimes of fast promoter kinetics and short initiation events produce a linear and super linear relationship between protein mean and variance respectively. (b) Illustration of promoter activation regimens dictated by fast promoter kinetics, short initiation events, and slow bursty kinetics. In each plot, the x-axis indicates time and the y-axis indicates promoter activity. Purple points and bars represent short or extended period of promoter activation. In the case of fast promoter kinetics, the transition between active and inactive is so rapid that the activation is approximated as constant.(c) Protein mean-variance relationship in a synthetic promoter library dataset (Mogno *et al* 2010) in log-log plot.

Figure S3: Description and results of the experimental validation. (a) Experimental design: We selected 15 genes that acquired a nucleosome when grown in YPEtOH compared to YPD using genome-wide nucleosome occupancy data. A control set of equal size was also built with genes with stable nucleosomes across the two conditions. For each gene in each set, we grew a corresponding GFP-fusion S. cerevisiae strain in YPD and YPEtOH to log phase and measured single-cell protein levels by flow-cytometry. (b) Representative results of 3 yeast strains from the test group. For each strain, the distribution of fluorescence intensity is shown in YPD (cyan) and YPEtOH (purple) respectively. The amount of residual variance (labeled as MIV or mean-independent variance) is displayed under each histogram. (c) Same as in (b) but for representative strains from the control group.

4 Supplementary references

- J. Paulsson. Models of Stochastic Gene Expression. *Phys. Life Rev.* **2** 157-75 (2005)
- Newman JR *et al.* Single-cell proteomic analysis of S. cerevisiae reveals the architecture of biological noise. *Nature* 2006 441(7095):840-6
- C. Miller *et al.* Dynamic transcriptome analysis measures rates of mRNA synthesis and decay in yeast. *Molecular and Systems Biology* **7** 458 (2011)
- S. Ghaemmaghami *et al.* Global analysis of protein expression in yeast. *Nature* **425** 737-41 (2003).
- F. Vallania *et al.* High-throughput discovery of rare insertions and deletions in large cohorts. *Genome Research* **20** 1391171397 (2010).
- R. Skupsky *et al.* HIV Promoter Integration Site Primarily Modulates Transcriptional Burst Size Rather Than Frequency.*PLOS Computational Biology* (2010)
- I. Mogno *et al.* TATA is a modular component of synthetic promoters. *Genome Research* **20** 1711171718 (2010).
- J.R. Chubb *et al.* Transcriptional pulsing of a developmental gene. *Current biology* **10** 1018171025 (2006).
- Hastie, Tibshirani, Friedman. The Elements of Statistical Learning. *Springer Books* (2009).
